# The multifaceted Foxp3^fgfp^ allele enhances spontaneous and therapeutic immune surveillance of cancer in mice

**DOI:** 10.1002/eji.201948251

**Published:** 2019-11-27

**Authors:** José Almeida‐Santos, Marie‐Louise Bergman, Inês Amendoeira Cabral, Vasco Correia, Íris Caramalho, Jocelyne Demengeot

**Affiliations:** ^1^ Instituto Gulbenkian de Ciência Oeiras Portugal

**Keywords:** anti‐CTLA4, hypomorph, regulatory T cells

## Abstract

It is well established that therapeutic impairment of Foxp3^+^ Treg in mice and humans favors immune rejection of solid tumors. Less explored is the impact Foxp3 allelic variants may have on tumor incidence, progression and therapy. In this work, we tested and demonstrate that the Foxp3^fgfp^ reporter allele, found previously to either enhance or reduce Treg function in specific autoimmunity settings, confers increased anti‐tumor immunity. Our conclusions stem out of the analysis of three tumor models of different tissue origin, in two murine genetic backgrounds. When compared to wild type animals, mice carrying the Foxp3^fgfp^ allele spontaneously delay, reduce or prevent primary tumor growth, decrease metastasis growth, and potentiate the response to anti‐CTLA4 monotherapy. These findings suggest allelic variances at the Foxp3 locus may serve as predictive indicators for personalized therapy and prognostics, and point at possible new therapeutic targets.

## Graphical abstract

Genetic alterations at the Foxp3 locus are most commonly associated with autoimmunity and inflammatory manifestations. In this work we evidence that a Foxp3 variant known to affect regulatory T cell functions acts as an hypomorph in a tumor context and enhances the effectiveness of anti‐CTLA4 immunotherapy.

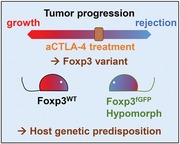

## Introduction

Regulatory T cells (Treg), a subset of CD4^+^ cells, express the transcription factor Foxp3 that defines a transcriptional profile essential for their differentiation and function 1. By controlling the activation of conventional T cells, Treg guarantee the establishment and maintenance of immune tolerance to self‐components 2. It is also well established that depletion or inhibition of Treg in mice and humans favors immune rejection of solid tumors 3, 4. Several human Foxp3 variants have been associated with various autoimmune diseases 5 and loss of function mutations is responsible for the fatal IPEX syndrome 6. Foxp3 allelic variants were also associated with increased susceptibility to colorectal and non‐small cell lung cancer, and progression of breast cancer 5. However, Foxp3 expression is not restricted to Treg and acts as a cell intrinsic tumor suppressor in solid tumors 7. Thus, it remains unclear whether allelic variants of the Foxp3 gene can affect immune surveillance of cancer. In turn, it is conceivable that protective Foxp3 alleles may also enhance the effectiveness of immunotherapies for cancer.

The development of Foxp3 reporters in mice fortuitously generated Foxp3 alleles which are functionally impaired, to various degrees 8, 9, 10, 11. The commonly used Foxp3^fgfp^ knock‐in allele, which encodes a Foxp3 protein fused at its *N*‐terminus to the enhanced green fluorescence protein (GFP), moderately alters the transcriptional signature and phenotype of Treg. The reported functional impact of the Foxp3^fgfp^ allele on Treg activities in vivo ranges from (i) impairment (in the NOD T1‐Diabetes model, scurfy or scurfy like diseases as well as an infection setting 9, 10, 11, 12, 13), (ii) neutrality (in the reference C57Bl/6 and BALB/c strains, an EAE model and the control of lymphopenia induced proliferation 10, 12, 14, 15), to (iii) enhancement (in the K/BxN arthritis model 9). Given these diverse outcomes, the impact of the Foxp3^fgfp^ allele on tumor immune surveillance remained to be tested. To dissociate tumorigenesis from anti‐tumor immunity, we used three transplantable tumor models. We evidence reduced primary tumor growth associated with increased immune responses, reduced metastatic progression, and enhanced response to anti‐CTLA4 monotherapy in Foxp3‐fGFP mice when compared to WT controls.

## Results and Discussion

### The Foxp3^fgfp^ allele enhances spontaneous immune‐surveillance of primary tumors

**Figure 1:**
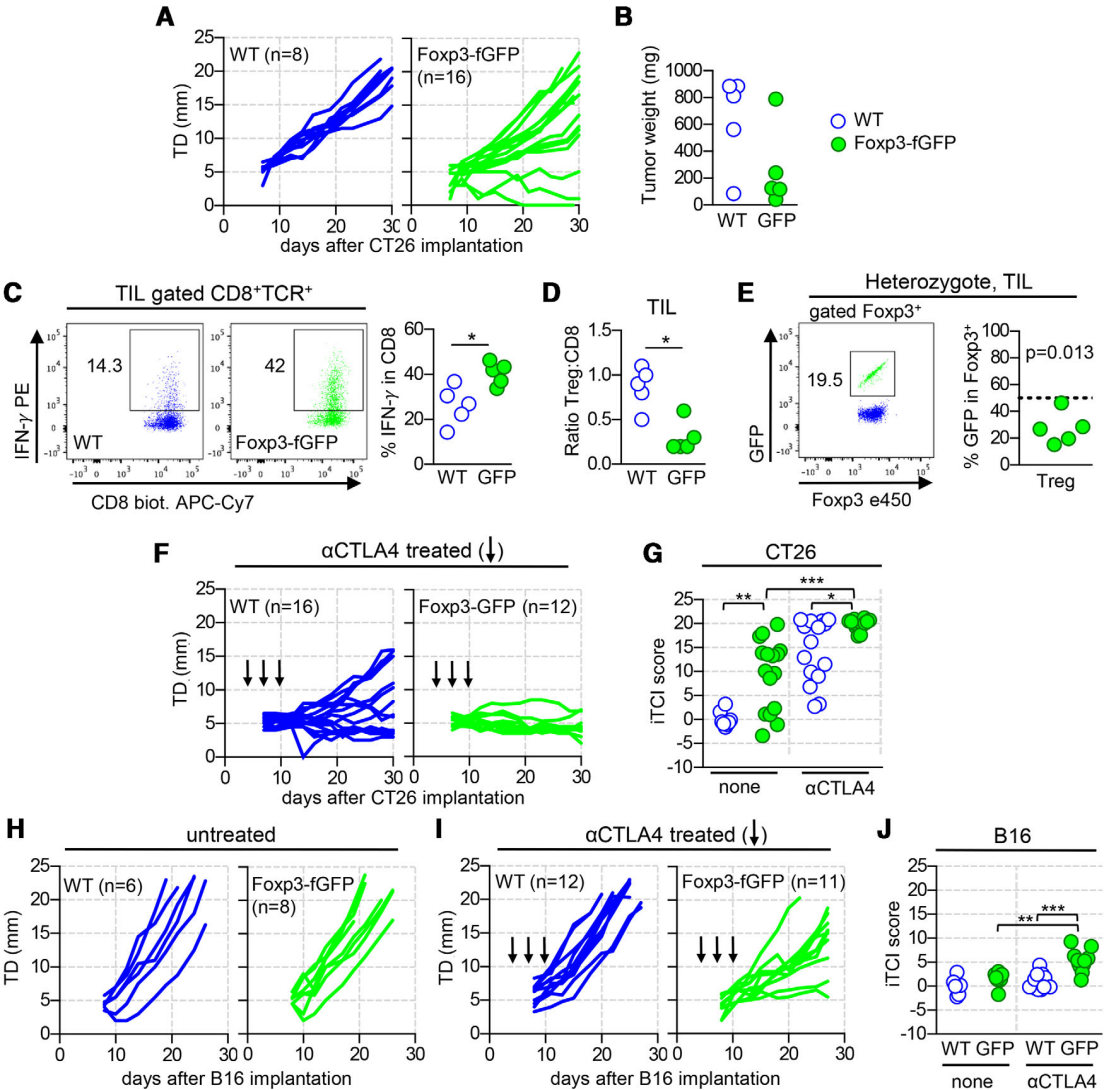
The Foxp3^fgfp^ allele promotes spontaneous and therapeutic tumor immune surveillance. 3×10^5^ CT26 (A–G) or 2×10^5^ B16 cells (H–J) were injected s.c. in the flank of mice with a BALB/c or B6 genetic background, respectively. Each line or each dot represents an individual mouse. Blue WT; green Foxp3‐fGFP. (A) Tumor diameter (TD) along time in WT (left) and Foxp3‐fGFP (right) littermates. Pool of three independent experiments for GFP (*n* = 4–6) and one matching WT (*n* = 8) mice, *p* = 0.0052. (B–D) Untreated mice 15 days post‐implantation analyzed for tumor weight (B) and tumor infiltrating lymphocytes (C–D), (*n* = 5, one experiment). (C) Representative flow cytometry‐plot for IFN‐γ in gated TCRβ^+^ CD8^+^ cells (left) and frequencies (right). (D) Ratio of Foxp3^+^ TCRβ^+^ CD4^+^ (Treg) to TCRβ^+^ CD8^+^. (E) Frequency of GFP expressing Treg in tumor infiltrating lymphocytes of Foxp3^fgfp/wt^ mice 15 days postimplantation. Representative FACS plot gated Foxp3^+^ TCRβ^+^ CD4^+^ (left) and frequencies (right) (*n* = 5, one experiment). (F) as in (A), except mice received 100 µg of aCTLA4 at day 4, 7, and 10 (vertical arrows) post‐tumor implantation. Pool of two independent experiments *n* = 4–10, *p* = 0.0449. (G) Individual Tumor Control Index (iTCI) scores for CT26 growth curves presented in (A and F). (H and I) As in (A) and (F) for B16 tumors and mice on a B6 background. Pool of two independent experiments for Foxp3‐fGFP (n = 4–7 mice) and one (untreated) or two (treated, *n* = 6) matching WT mice, *p* = 0.0041. (J) iTCI scores for B16 growth curves shown in (I) and (J). Statistical analysis used nonparametric Mann–Whitney test (*p < 0.05, **p < 0.005, and ***p < 0.001) in (B, D, E, G, and J), one sample *t*‐test in (E) and two‐way ANOVA (*p* value inserted in the legend) for (A, F, H, and I).

**Figure 2:**
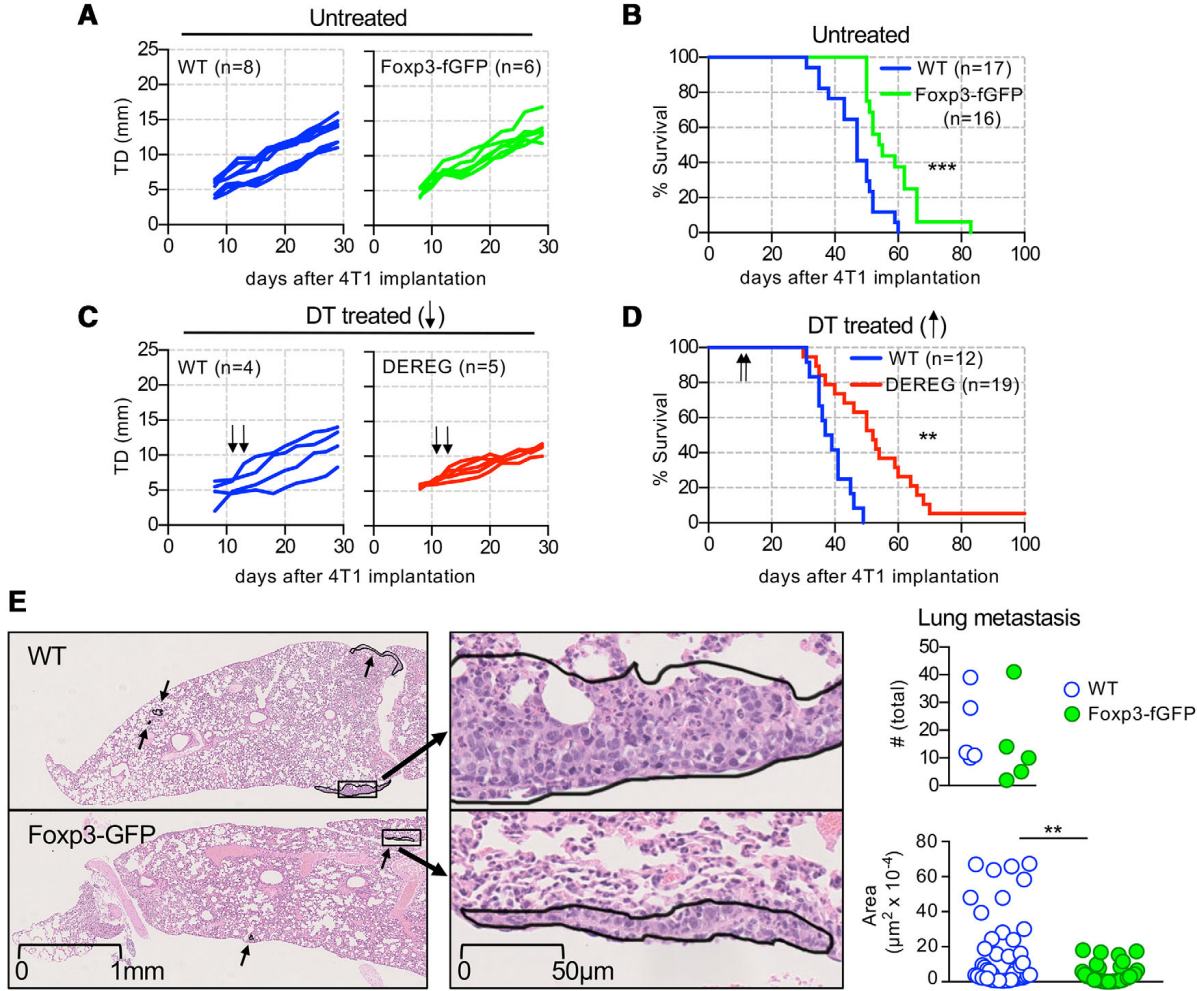
The Foxp3^fgfp^ allele reduces metastasis progression. Mice on a BALB/c background were injected s.c. with 5 × 10^4^ 4T1 cells. (A and B) Tumor growth (A), one experiment, and mice survival (B), pool of two independent experiments *n* = 6–10, in WT and Foxp3‐fGFP mice. (C and D) Tumor growth (C), one experiment, and survival (D), pool of two independent experiments *n* = 5–14, in WT and DEREG littermates treated with DT (arrows). (E) Left, representative histological analysis of the lungs from WT and Foxp3‐fGFP mice 24 days postimplantation. Small arrows point at metastatic foci, long arrows indicate areas presented at high magnification. Right, metastasis quantification and area estimates. For the latter, plotted is the individual area of each metastatic focus identified in five mice, for each genotype. Representative of two independent experiments (*n* = 5–6 mice). Statistical analysis used two‐way ANOVA for tumor growth (not significant for both A and C), Logrank test for survival curves, and Nonparametric Mann–Whitney test for metastasis number and area. **p < 0.005 and ***p < 0.001.

To test whether the Foxp3^fgfp^ allele provides enhanced anti‐tumor immunity we first chose to monitor the CT26 colorectal carcinoma cell line, derived from a BALB/c (Ba) mouse, which intrinsic immunogenicity is readily revealed upon Treg depletion 16. While CT26 cells engraft and grow vigorously in syngeneic WT animals, the tumor is consistently and fully rejected upon administration of diphtheria toxin (DT) in DEREG mice, either one 16 or two (Supporting Information Fig. 1) weeks after implantation. Of note, in this experimental setting as in others, treatment of WT mice with DT is not innocuous (compare left panels Supporting Information Fig. 1A, Figs. 1A, 2A and 2C).

We generated Ba.Foxp3‐fGFP mice which, at steady state and compared to gender and age matched WT animals, have slightly underrepresented Treg expressing increased Foxp3 protein (as reported for mice on the B6 background 9), bear normal numbers of activated or IFN‐γ producing T cells (Supporting Information Fig. 2) and do not show sign of disease. Strikingly, upon implantation of CT26 (Fig. 1A), tumor growth was either delayed or fully prevented in more than a third of the Foxp3‐fGFP mice, while it was regular in WT controls. Due to the heterogeneous shape of each tumor growth curve, we relied on the individual Tumor Control Index 17, which informs on tumor regression, stability, and rejection, to infer more faithful statistical analysis (Fig. 1G). To ascertain that immune responses were enhanced in Foxp3‐fGFP mice, tumor infiltrating lymphocytes were analyzed 15 days post‐implantation (Fig. 1B–D and Supporting Information Fig. 3). Compared to WT mice, the tumor weight was reduced in most Foxp3‐fGFP animals (Fig. 1B), Treg frequency, and number as well as CD4 cell activation were similar (Supporting Information Fig. 3), yet the frequency of CD8 IFN‐γ producing cells was increased (Fig. 1C) and the ratio Treg to CD8 was decreased (Fig. 1D), two hallmarks of anti‐tumor adaptive response. In the tumor draining lymph node, these differences were not observed (Supporting Information Fig. 3). These data suggest the Foxp3‐fGFP protein affects Treg ability to dampen CD8 responses rather than Treg stability and dedifferentiation into effector CD4 cells, or their migration capacity.

We next analyzed Foxp3^wt^ and Foxp3^fgfp^ expressing Treg in heterozygous Foxp3^fgfp/wt^ females (Fig. 1E, Supporting Information Figs. 2 and 4), which are mosaic, owing to the fact that Foxp3 is located on the X chromosome. While Foxp3^wt^ and Foxp3^fgfp^ Treg were equally represented in spleen and LN at steady state (Supporting Information Fig. 2), Foxp3^fgfp^ Treg were largely underrepresented in tumor infiltrating lymphocytes and in most draining lymph node 15 days post‐implantation of CT26 (Fig. 1E and Supporting Information Fig. 4). Similar cellular disadvantage was previously reported in B6.Foxp3^fgfp/wt^ females implanted with the B16 melanoma, although the impact of the Foxp3^fgfp^ allele on tumor growth was not addressed 10. Foxp3^wt^ and Foxp3^fgfp^ Treg expressed the same level of Foxp3 protein at steady state and in tumor bearing heterozygous animals (Supporting Information Figs. 2 and 4), suggesting the overexpression observed in homozygous animals (Supporting Information Fig. 2) is not a cell intrinsic property. Finally, Foxp3^fgfp^ Treg display increased CTLA4 and CD25 expression levels, possibly a signature of enhanced IRF4 pathway 9 or compensatory cellular overactivation 12.

We conclude that the Foxp3^fgfp^ allele potentiates spontaneous anti‐tumor immunity without affecting health in the BALB/c reference strain. This finding resonates with the recent evidence that a single alanine replacement in the N‐terminal proline‐rich region of Foxp3, where no specific motif has been identified, does not affect health at steady state, and reduces growth of the MC38 primary tumor 18.

### The Foxp3^fgfp^ allele enhances the effectiveness of cancer immunotherapy

We next tested whether the Foxp3^fgfp^ allele potentiates therapeutic response to anti‐CTLA4 (aCTLA4) treatment. We administered aCTLA4 during the first week following tumor implantation in WT and Foxp3‐fGFP mice, and followed subsequent tumor progression (Fig. 1F‐J). As previously reported 19, WT mice implanted with CT26 partially responded to aCTLA4 monotherapy, with only a fraction of them rejecting or delaying tumor growth. In contrast, all treated Foxp3‐fGFP mice prevented tumor growth, with most animals maintaining a tumor free state 1‐month post‐implantation (Fig. 1F and G). These dramatic results encouraged us to test B6.Foxp3‐fGFP mice implanted with the poorly immunogenic B16 melanoma for which aCTLA4 monotherapy has been reported ineffective 19. Strikingly, although the Foxp3^fgfp^ allele by itself did not affect B16 engraftment or progression (Fig. 1H), aCTLA4 treatment delayed tumor growth in the majority of Foxp3‐fGFP mice but not in WT controls (Fig. 1I and J). Finally, enhanced therapeutic response provided by the Foxp3^fgfp^ allele was not accompanied by overt systemic effects as indicated by constant body weight (Supporting Information Fig. 5). Our finding that the Foxp3^fgfp^ allele potentiates aCTLA4 therapy, together with the evidence that aCTLA4 kills Treg 20, 21, echoes with a previous report indicating the same allele amplifies the effectiveness of DT treatment in DEREG mice to induce severe autoimmunity 12, as a consequence of defective Treg repopulation. We also note that in heterozygote mice, Foxp3^fgfp^ Treg express higher levels of CTLA4 than WT cells (Supporting Information Figs. 2 and 4), which could potentially facilitate aCTLA4 mediated killing of Treg.

### The Foxp3^fgfp^ allele delays metastasis dissemination

The last phase of tumor progression is metastatic dissemination, a process well modelled by the 4T1 breast carcinoma derived from a BALB/c mouse. A moderate role for Treg in facilitating 4T1 primary tumor growth 16 and precipitating death 22 has been reported. Monitoring WT and Foxp3‐fGFP animals implanted subcutaneously with 4T1 cells revealed similar growth of the primary tumor while survival was significantly prolonged in the latter group (Fig. 2A and B and Supporting Information Fig. 6). Prolonged survival was also observed in DEREG animals administrated with DT, as a mean to induce a transient depletion of Treg, early after 4T1 implantation (Fig 2C and D). We next ascertained the prolonged survival in Foxp3‐fGFP mice bearing 4T1 tumors associated with reduced metastasis. We first confirmed that resection of the primary tumor during the second week post‐implantation, but not later, greatly enhances survival (Supporting Information Fig. 6), an intervention shown by others to prevent the metastatic process 23. This result suggested analysis at 3 weeks post implantation would be suitable to quantify metastasis dissemination in WT and Foxp3‐fGFP mice. Lungs were harvested and a histological assessment of metastasis number and size was performed (Fig. 2E). Although the number of metastatic foci was similar in both groups of mice, large nodules were only found in WT animals. Together, these findings indicate the Foxp3^fgfp^ allele restrains the dissemination stage of 4T1 tumors.

## Concluding remarks

Of relevance for experimental biology at large, our work provides further evidence that experimental variations can be related to reporter alleles, now in the context of cancer immune surveillance. In the frame of tumor immunotherapy, previous indications that the introduction of GFP at the N‐terminal part of Foxp3 affects its interaction with HIF1‐alpha and IRF4 9, HDAC7, Tip60 and Eos 10, or PRC2 12 open the possibility that identification of peptides interfering specifically with either of these interactions could provide novel therapeutic tools, as is already developed for Foxp3 C‐terminal part‐NFAT interactions 24. Finally, although mice are unlikely to be faithful models of humans continuously exposed to inflammatory triggers, it is conceivable that weak human Foxp3 hypomorphs on an otherwise healthy genetic background would be beneficial to the host when fighting cancer, without tilting the balance toward autoimmunity. The identification of such human Foxp3 variants may guide cancer prognostics and therapeutic strategies.

## Materials and Methods

### Mice

C57BL/6 Foxp3^tm2Ayr^ were backcrossed for at least 10 generations to the BALB/c.ByJ background (Ba.Foxp3‐fGFP). BALB/c‐Tg(Foxp3‐DTR/EGFP)23.2Spar (DEREG) were maintained as hemizygotes. All mice were bred and raised at the Instituto Gulbenkian de Ciência under specific pathogen‐free conditions. Experiments were conducted according to the Federation of European Laboratory Animal Science Association guidelines and approved by the ethic committee of the IGC. Where indicated, mice were injected subcutaneously in the flank with 3 × 10^5^ CT26 (ATCC), 2 × 10^5^ B16‐F10‐luc2 (B16) (CaliperLS), or 5 × 10^4^ 4T1 (ATCC) cells, and intraperitoneally with 1 µg DT (322326‐1, Calbiochem), or 100 µg of mAb 4F10 (aCTLA4). Tumor size was measured with a caliper every 2 or 3 days, from day 8 post injection, and tumor diameter (TD) calculated as TD = (L + W)/2. For ethical reasons, mice were sacrificed when TD ≥ 20 mm.

### Antibodies and FACS analysis

Tumor infiltrating lymphocytes were recovered after tumor digestion for 30 min in HBSS (Life Technologies) containing 10 mM EDTA, 0.1% BSA, 1 mg/mL collagenase type IV and 100 µg/mL DNAse I, followed by separation on a Percoll gradient (all from Sigma). For cytokine analysis, cells were incubated for 4 h at 37ºC with PMA and Ionomycin (both Sigma), and Brefeldin A (eBioscience). Cells were first pre‐incubated with Fc‐block, stained for surface markers, and for intranuclear and intracytoplasmic staining, incubated overnight at 4ºC in fix/permeabilization buffer (eBioscience). mAb are listed in Supporting Information Table 1. Samples were analyzed on Cyan ADP or BD LSRFortessa™ X‐20 instruments and with the FlowJo software, following EJI guidelines 25 and gating strategies as in Supporting Information Fig. 7.

### Histological assessment

Mice were perfused with PBS and lungs fixed in 10% formalin. For each animal, one every 100 paraffin sections (5 µm thick, ∼20 per sample) were stained with H&E. Metastasis foci and areas were determined by the Histopathology Unit at IGC.

### Statistical analysis

The *individual* Tumor Control Index (iTCI) was derived from the TCI 17 which compiles three scores per experimental group assessing tumor inhibition, stability, and rejection. We modified this method to calculate the three sub‐scores for each individual mouse (iTCI), an improvement which allows for statistical analysis between experimental groups. Statistics of iTCI and cellular analysis were performed using nonparametric Mann–Whitney test. Tumor growth was also analyzed using two‐way ANOVA. Log‐rank tests were used for survival curves and two‐way ANOVA for body weight kinetics. Correlation analyses were performed using Pearson correlation coefficients.

## Conflict of interest

The authors declare no commercial or financial conflict of interest.

**Abbreviation**
DT: diphteria toxin

## Supporting information

Supplementary material
