# Supplementary material for: The multifaceted Foxp3fgfp allele enhances spontaneous and therapeutic immune surveillance of cancer in mice
Source: Eur J Immunol. 2019 Nov 27;50(3):439–44. doi: 10.1002/eji.201948251 (PMC7078871; doi:10.1002/eji.201948251)
Supplement: Supplementary file 1 — Supplementary material [file EJI-50-439-s001.pdf]

European Journal of Immunology

Supporting Information
for
DOI 10.1002/eji.201948251

José Almeida-Santos, Marie-Louise Bergman, Inês Amendoeira Cabral,
Vasco Correia, Íris Caramalho and Jocelyne Demengeot

The multifaceted Foxp3^{fgfp} allele enhances spontaneous and therapeutic immune surveillance of cancer in mice

© 2019 The Authors. *European Journal of Immunology* published by WILEY-VCH Verlag GmbH & Co. KGaA, Weinheim

The multifaceted Foxp3^{fgfp} allele enhances spontaneous and therapeutic immune surveillance of cancer in mice

Supporting Information Figure S1

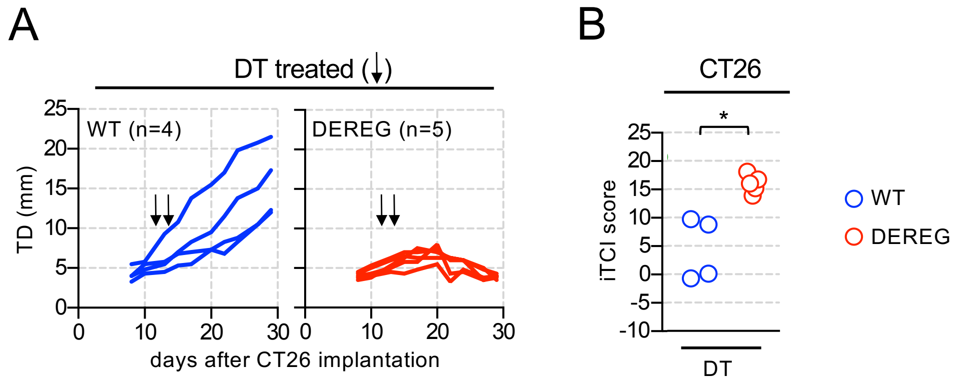

Figure S1. Control of CT26 growth by Treg. A) Tumor diameter (TD) along time in WT (left) and DEREG (right) littermates, treated with 1µg of diphtheria toxin (DT) at day 13 and 14 (arrows) post-implantation. One experiment, p=0.0196, two-way ANOVA. B) Individual Tumor Control Index (iTCI) scores, Mann-Whitney test., *P < 0.05

Supporting Information Figure S2

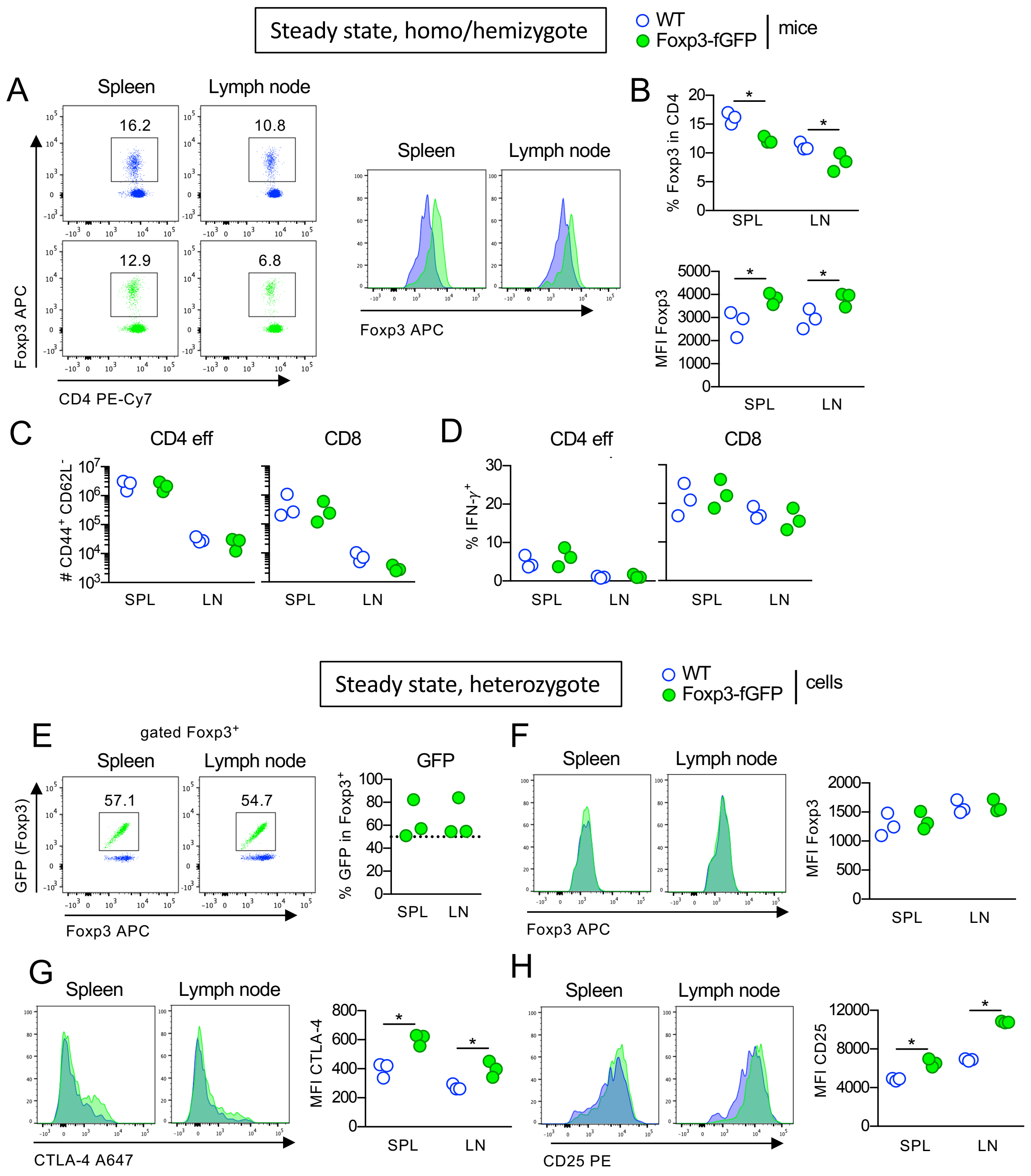

Figure S2. Mild cellular alterations at steady state in Ba.Foxp3-fGFP mice. Inguinal lymph nodes (LN) and spleen (SPL) from age matched Ba.WT and Ba.Foxp3-fGFP mice (**A-D**) or heterozygous Ba.Foxp3$^{fgfp/wt}$ (**E-H**) were analyzed by flow cytometry. **A, B**) Percentage of Foxp3$^+$ cells inside gated live CD4$^+$ TCR$^+$ cells and Foxp3 expression in gated Foxp3$^+$ cells. Representative FACS plot (A) and frequencies and mean fluorescence intensity (MFI) (B) representative of 2 independent experiments (n = 3 mice). **C, D**) Analysis of CD4 Foxp3- (CD4eff) and CD8 T cells. Absolute number of activated (CD44$^+$ CD62L$^-$) cells (C) and frequency of IFN-γ producers (D). One experiment. **E)** GFP expressing cells in gated Foxp3$^+$ cells, representative FACS plot (left) and frequencies (right). **F-H)** Foxp3 (F), CTLA4 (G) and CD25 (H) mean fluorescent intensity (MFI) in gated Foxp3$^+$ cells. Statistical analysis performed using nonparametric Mann-Whitney test. *P < 0.05, except in E using one-sample t test (not significant)

Supporting Information Figure S3

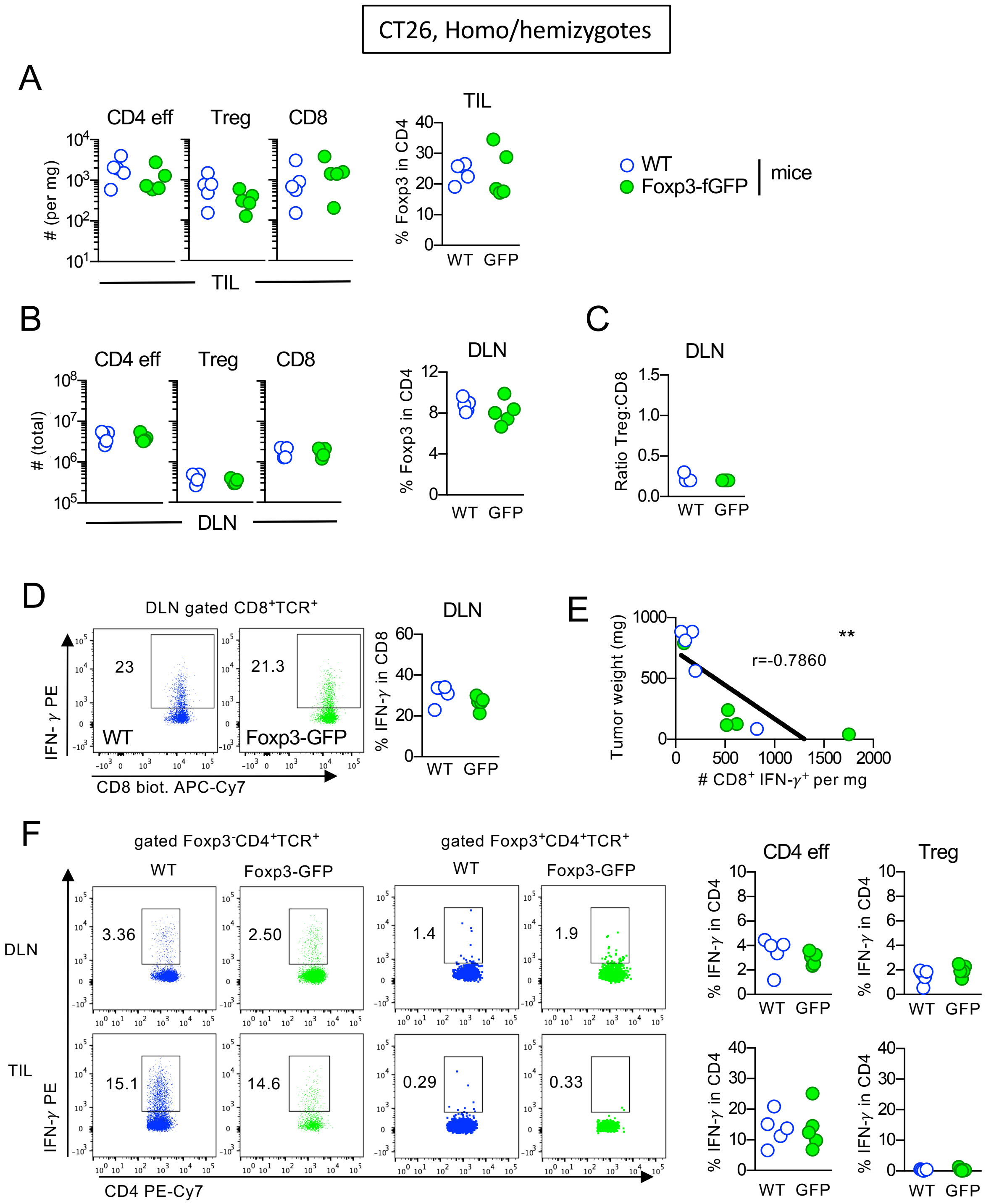

Figure S3. Complement to Figure 1C-E

Additional data to the cellular analysis shown in Fig1 C-E addressing Ba.WT and Ba.Foxp3-fGFP mice implanted with CT26 15 days earlier. The same mice were further analyzed. **A**) Numbers of TIL $CD4^+$ $Foxp3^-$ (CD4 eff), $CD4^+$ $Foxp3^+$ (Treg) and $CD8^+$ (CD8) T cells recovered per mg of tumor, and frequency of $Foxp3^+$ cells inside gated $CD4^+$ T cells. **B-D**) Analysis of tumor draining lymph nodes (DLN). Total number of $CD4^+$ $Foxp3^-$ (CD4 eff), $CD4^+$ $Foxp3^+$ (Treg) and $CD8^+$ (CD8) T cells, and frequency of $Foxp3^+$ cells inside gated $CD4^+$ T cells (B); Treg to CD8 ratio (C); frequency of IFN-γ producing CD8 cells (D). **E**) Correlation between tumor weight and number of TIL IFN-γ producing CD8 T cells per mg of tumor. **F**) Frequency of IFN-γ producing CD4 (gated $CD4^+$ TCR^+) and Treg (gated $CD4^+$ TCR^+ $Foxp3^+$) from TIL and DLN with representative FACS plots (left) and frequencies (right). Statistics were performed using nonparametric Mann-Whitney test, not significant. Correlation analysis (E) performed using Pearson correlation coefficients.

Supporting Information Figure S4

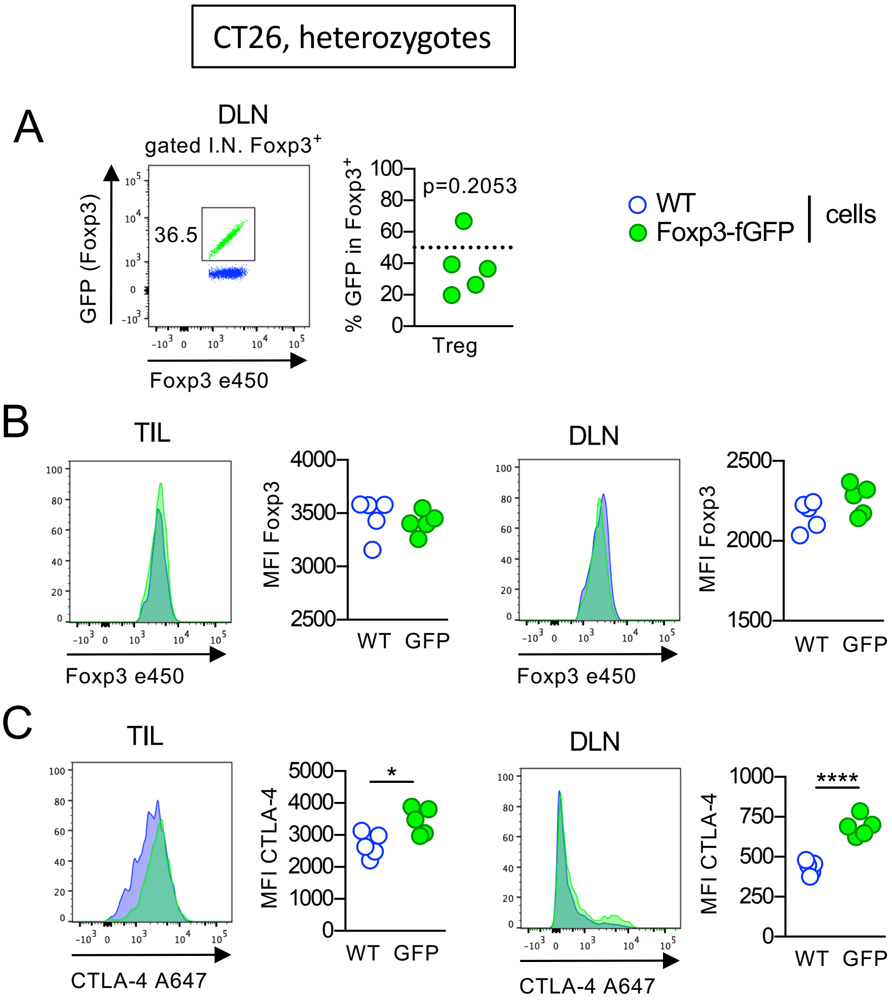

Figure S4. Complement to Figure 1F

Complement to the cellular analysis in Fig. 1F addressing heterozygote Ba.Foxp3$^{fgfp/wt}$ mice implanted with CT26 15 days earlier. The same mice were further analyzed. **A**) Frequency of GFP expressing cells in gated Foxp3^{+} cells recovered from DLN. **B**) Mean fluorescent intensity (MFI) in gated Foxp3^{+} cells from TIL and DLN for Foxp3 (B) and CTLA4 (C). Statistics were performed using one-sample t test (A) , nonparametric Mann-Whitney test (B), two tailed parametric T test, (C), *P < 0.05; ****P < 0.0001

Supporting Information Figure S5

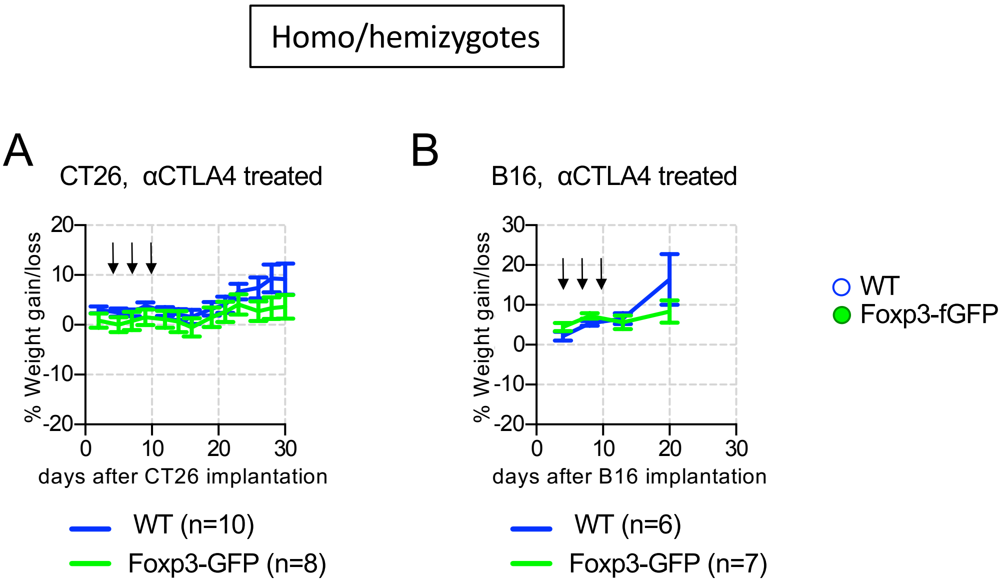

Figure S5. Complement to Figure 1G and J
Body weight along time in aCTLA4 treated (arrows) WT and Foxp3-fGFP mice on the Ba (A) or B6 (B) background implanted with CT26 (A) or B16 (B) tumor. One of the 2 experiments pooled in Fig. 1G and J. Shown are mean with SEM. Statistics performed using two-way ANOVA, not significant.

Supporting Information Figure S6

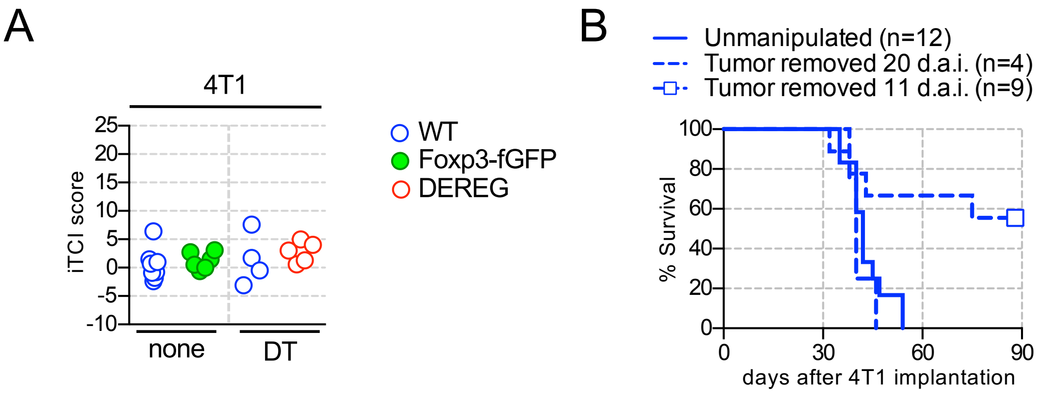

Figure S6. Complement to Figure 2.
A) iTCI scores for 4T1 growth curves shown in Fig. 2A and C. Statistical analysis performed using nonparametric Mann-Whitney test, not significant. **B**) Survival of WT mice submitted to primary tumor resection (doted lines) or not (plain line) at days 11 and 20 after 4T1 implantation (d.a.i.), one experiment.

Supporting Information Figure S7

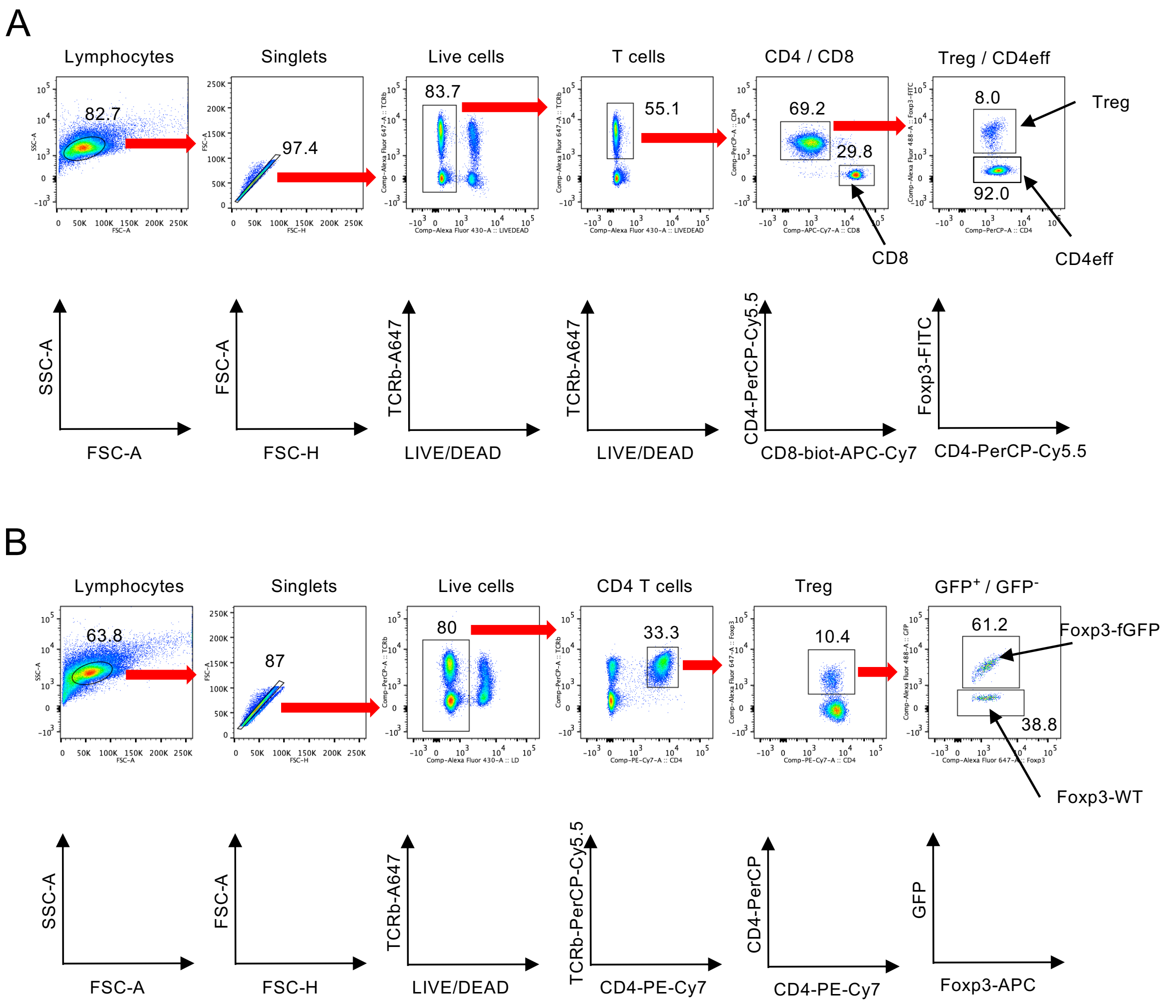

Figure S7. Gating Strategy for flow cytometry analysis. **A**) Sequential gating to identify CD8, CD4eff (Foxp3$^-$), and Treg. Arrows in the last 2 panels on the right point at the gates used for further phenotyping of each cell subset, e.g for CD8 T cells producing IFN-γ in Fig 1D. **B**) Sequential gating to identify GFP expressing Treg in heterozygote Foxp3$^{fgfp/wt}$ mice. The gates in the last panel on the right are those used for further phenotyping of each cell subset., e.g. for CTLA4 expression level in FigS1G. LIVE/DEAD® Fixable Yellow Dead Cell Stain, for 405 nm excitation (Molecular Probes®), was purchased from Invitrogen™.

Table S1. monoclonal Antibodies used in this study

**Reactivity**	**Clone**	**Conjugate**	**Supplier**
CTLA4	4F10	none	In house**
Fc (Fc-block)	2.4G2	none	In house
CD4	GK1.5	PerCP-Cy5.5, PE-Cy7	BioLegend
CD8	YTS169.4	Biot/PB	In house
CD25	PC61	PE	In house
CD44	IM7	FITC	BD Pharmingen
CD44	IM7	e450	eBioscience
CD62L	H1.2F3	Biot<br>PE	BioLegend
CD152 (CTLA4)*	4F10	A647	In house
Foxp3*	FJK-16s	FITC<br>e450/<br>APC	eBioscience
INF-γ*	XMG1.2	PE	BD Pharmingen
TCR-β	H57-597	PerCP-Cy5.5	eBioscience
TCR-β	H57-597	A647	In house
Streptavidin		APC-Cy7	BioLegend

APC, allophycocyanin; Biot, biotin; Cy, cychrome; PB, Pacific Blue; PE, phycoerythrin; FITC, fluorescein isothiocynate; e, eFluor; PerCP, Peridinin-Chlorophyll-protein

* intracellular/intranuclear staining

**All antibodies produced in house were purified on protein G columns. When indicated Ab were also conjugated in house
